# The association between dietary total antioxidant capacity and odds and severity of irritable bowel syndrome among Iranian adults: a cross-sectional study

**DOI:** 10.1186/s12876-022-02531-3

**Published:** 2022-11-19

**Authors:** Solaleh Saneie, Azadeh Aminianfar, Farzad Shidfar, Ammar Hassanzadeh Keshteli, Ahmad Esmaillzadeh, Peyman Adibi

**Affiliations:** 1 Department of Nutrition, School of Public Health, Iran University of Medical Sciences, Tehran, Iran; 2 Research Center for Biochemistry and Nutrition in Metabolic Diseases, Kashan University of Medical Sciences, Kashan, Iran; 3 Department of Medicine, University of Alberta, Edmonton, Canada; 4 Department of Community Nutrition, School of Nutritional Sciences and Dietetics, Tehran University of Medical Sciences, P.O. Box 14155-6117, Tehran, Iran; 5 Obesity and Eating Habits Research Center, Endocrinology and Metabolism Molecular-Cellular Sciences Institute, Tehran University of Medical Sciences, Tehran, Iran; 6 Department of Community Nutrition, School of Nutrition and Food Science, Isfahan University of Medical Sciences, Isfahan, Iran; 7 Integrative Functional Gastroenterology Research Center, Isfahan University of Medical Sciences, Isfahan, Iran

**Keywords:** Dietary total antioxidant capacity, dTAC, Irritable bowel syndrome, IBS, Cross-sectional

## Abstract

**Background:**

Little evidence is available in terms of the role of dietary antioxidants in the management of irritable bowel syndrome (IBS) disease. This study aimed to examine the association between dietary total antioxidant capacity (dTAC) and odds of IBS and its severity.

**Methods:**

This cross-sectional study was conducted on 3,362 Iranian adults who were referred to health centers in Isfahan province, Iran. Participants' dietary intakes were collected using a semi-quantitative validated food frequency questionnaire (DS-FFQ). The dTAC was measured by the ferric-reducing antioxidant power (FRAP) method. Multivariable binary or ordinal logistic regression analyses were performed to estimate any associations between dTAC and odds of IBS, IBS severity, and IBS subtypes.

**Results:**

The average age and BMI of the participants and dTAC score were 36.3 ± 7.87 year, 24.9 ± 3.82 kg/m2. The prevalence of IBS, IBS with constipation (IBS-C), IBS with diarrhoea (IBS-D), mixed IBS (IBS-M), and un-subtyped IBS (IBS-U) were 22.2, 7.5, 4.6, 3.8, and 6.2%, respectively. In crude and adjusted models, the results did not show any significant association between dTAC and odds of IBS among whole and gender-age stratified populations. Being in the third compared with the first tertile of dTAC was not also significantly associated with odds of IBS severity. Besides, there were no significant associations between dTAC and odds of IBS-C, IBS-D, IBS-M, and IBS-U.

**Conclusion:**

This study indicates that dTAC may not be associated with the odds of IBS and its severity even after stratification for gender and body mass index.

## Introduction

Irritable bowel syndrome (IBS) is a highly costed and potentially disabling functional gastrointestinal (GI) disorder that affects 11.2% of populations globally [1]. While there is no documented information about its prevalence in Iran, the results of a cross-sectional study showed that 15% of its population was affected by IBS [2]. Predominant symptoms of this disorder are difficulty in defecation and abdominal pain which may increase with stress [3]. Studies suggest inflammation or injury to tissues as potential causes for the development of symptoms in IBS [4]. The disease is associated with increased pro-inflammatory cytokines, according to previous research [5, 6]. In IBS, the antioxidant defense system appears to be impaired, which may also contribute to its pathogenesis [7].

Patients with IBS are treated with several different pharmacological therapies, however, most prefer to avoid medications and prefer alternative therapies [8]. Research has investigated habitual dietary intakes in patients with IBS and has found contradictory results. In some studies, patients with IBS compared with healthy controls consumed significantly less protein, fibre, calcium, and beta carotene [9–11]. In addition, a study found carbohydrate-rich foods, fatty foods, coffee, and spicy foods most frequently caused IBS symptoms [12]. In contrast, other studies found no significant differences between patients with IBS and control groups in macronutrient, micronutrient, or food group intake [13, 14]. Nevertheless, there are limited data regarding the overall quality of their habitual diet in IBS patients. A case–control study showed IBS patients had lower dutch healthy diet scores than controls. The study found that patients' diets were lower in fibre and fructose, but higher in total fat and added sugars [15]. Other epidemiological studies also assessed the associations of dietary indices, as new tools for the prediction of the associations between dietary habits and disease risk, concerning IBS risk [16–19], however, there is little about the potential role of other components of a diet such as dietary antioxidants in this context. Due to interactions between nutrients and synergetic effects of antioxidants in a diet, recent nutritional studies estimate the overall amount of antioxidants in a diet using the dietary total antioxidant capacity (dTAC) index [20]. This index shows the overall capacity of antioxidants in food to protect against free radicals [21]. Also, it can be regarded as an indicator of diet quality [21] as it was positively associated with other dietary quality indices such as Healthy Eating Index, Mediterranean Diet Score, and Diet Quality index [22, 23]. Moreover, studies show that dTAC is a good predictor of plasma antioxidant status [24–26]. It has been indicated that dTAC has been inversely associated with plasma levels of high-sensitivity C-reactive protein [27] and plasma malondialdehyde [28], and stress [29] as major risk factors for developing IBS symptoms. In this line, there have been several studies conducted to confirm a possible link between chronic diseases and dTAC, but the results have been contradictory. Accordingly, this index was inversely associated with the risk of breast cancer [30], prostate cancer [31], and GI cancers [32]. In addition, it decreased the odds of non-alcoholic fatty liver disease [33] and prediabetes [34] in two case–control studies. In contrast, a study found an increased obesity risk in women with higher dTAC scores [35]. In other observational studies, there was no association between dTAC and renal function and chronic kidney disease progression [36], breast cancer risk [37], and waist circumference [35].

Overall, dietary antioxidants seem to have an inevitable role in the pathogenesis of GI disease, however, only one study has addressed such an important issue [38]. Also, Iranians are believed to be undergoing a nutritional transition from healthy to unhealthy diets (massive meals, high refined grain consumption, high carbohydrate consumption, and hydrogenated oil consumption) which may contribute to such chronic diseases [39]. Therefore, we performed this study to evaluate the association between DTAC and odds and severity of IBS symptoms among the Iranian population.

## Materials and methods

### Participants

The SEPAHAN (Epidemiology of Psychological, Alimentary Health, and Nutrition) project was used as a source of data for this cross-sectional study [40]. The project mainly aimed to find out whether lifestyle and psychological factors have an association with functional GI disorders in adults (18–55 years) in Isfahan province. The SEPAHAN project included non-academic staff, managers and their socio-economic status, and employees who were working in fifty healthcare centers affiliated with Isfahan University of Medical Sciences (IUMS). A total of 10,087 subjects were enrolled to complete self-administered questionnaires in phase one of the project. These questionnaires were used to collect sociodemographic data, anthropometric measurements, medical history, physical activity levels, and dietary intake data. In this phase, 86.16% of the questionnaires were returned, with 8.691 subjects completing them. In the second phase, participants were asked to fill out a questionnaire about their gastrointestinal profile (64.6% of participants provided information about GI health (N = 6239)). As a result of combining the data from both phases, we had information on 4,763 subjects' dietary intakes and GI disorders from phases one and two. Finally, we excluded participants in the final analyses if their daily energy intakes were lower than 800 kcal/d or upper than 4200 kcal/d. After this exclusion, 3,362 participants remained for the final analysis. Isfahan University of Medical Sciences Bioethics Committee approved the project protocol (Approval No. 189069, 189082, and 189086). All methods were carried out under relevant guidelines and regulations and all subjects filled out a written informed consent form before participating.

### Dietary intakes assessment and dTAC calculation

We gathered the dietary intakes of the study participants using a validated 106-item dish-based semi-quantitative food frequency questionnaire (DS-FFQ) [41]. The DS-FFQ included five categories of food and dishes, including mixed dishes (canned or cooked, 29 items), grains (different kinds of bread, potatoes, cakes, and biscuits, 10 items), fruits and vegetables (22 items), dairy products (dairies, cream, and butter, 9 items), and miscellaneous food items and beverages (including beverages, fast foods, sweets, nuts, and desserts, 36 items). Subjects reported their consumption of these 106 food items based on nine multiple-choice frequency response categories, ranging from "never or 1/month" to "12/day". Accordingly, 6–9 options were available for frequency responses. For foods consumed rarely, we removed the high-frequency category, while we added several multiple-choice categories for foods consumed frequently. According to the frequency of consumption of each food item, grams of each food item was estimated based on household measurements. In calculating DTAC, ferric-reducing antioxidant power values (mmol/100 g) were calculated for each food item in the DS-FFQ. The FRAP assay is used to measure the ability of total antioxidants in a diet that reduces ferric ions to ferrous ones [42]. Accordingly, we calculated the FRAP values for foods based on the previous research [43]. In the case of similar food items (e.g., a variety of bread, meats, etc.) or absence of the FRAP values, the values of the nearest comparable food were assigned. Then, a person's dTAC was calculated by multiplying each frequency consumption value by the FRAP value of each food item.

### Assessment of IBS

A version of the Rome III questionnaire, which was developed for the Iranian population, was utilized to measure IBS symptoms. [44]. Because most participants found it difficult to respond to an original questionnaire (never, 1 day per month, 1 day per month, 2–3 days per month, 1 day per week, more than 1 day per week, and every day), we used a questionnaire with a 4-item rating scale (never/rarely, sometimes, often, and always). Symptoms with long-term experiences (over six months) were replaced with shorter-term experiences (less than three months) [45]. The presence of at least two or more of the following criteria was required to diagnose subjects with IBS before the initiation of research (within the last three months); improvement in abdominal discomfort or pain with defecation sometimes and the onset of a such condition related to changes in stool frequency or form [46]. Constipation-predominant IBS was identified if they had hard or lumpy stools, as well as a lack of loose, mushy, or watery stools [47]. If they had watery stools and no firm stools regularly, they had diarrhea-predominant IBS [48]. In this study, subjects with hard or lumpy stools, or loose, mushy, or watery stools at least occasionally, were considered to have mixed IBS [49]. Other participants were considered as unshaped kinds of IBS. The severity of abdominal pain in the last three months was also reported by the subjects and classified as mild, moderate, severe, and very severe.

### Assessment of other lifestyle factors

We collected information on other lifestyle factors such as age, sex, smoking history, marital status, medication use (included omeprazole, pantoprazole, ranitidine, cimetidine, famotidine, clidinium, hyoscine, blandola, dimethicone, digestive, pancreatin, antiacid, diphenoxylate, loperamide, nortriptyline, amitriptyline or imipramine, fluoxetine, citalopram, fluvoxamine, and sertraline), and disease history (having chronic disease included diabetes and colitis) through a self-reported questionnaire. Anthropometric measurements including weight, height, waist circumference, and Body mass index (BMI) were also measured using standard methods. A pilot study on a sample of 200 participants found reasonable results for the usefulness of these self-reported anthropometric measures. The results showed statistically significant correlation coefficient for weight 0.95 (*P* < 0.001), height 0.83 (*P* < 0.001), WC 0.60 (*P* < 0.001), and BMI 0.70 (*P* < 0.001) from these self-reported values compared to the measured values [50]. The General Practice Physical Activity Questionnaire (GPPAQ) was completed by the subjects to assess physical activity levels. Accordingly, subjects were classified as physically inactive (< 1 h/week) and physically active (≥ 1 h/week). Also, intra-meal fluid intake (< 3 glasses/ ≥ 3 glasses), meal regularity (often or frequently or always and never or rarely), and chewing efficiency (a lot/not a lot) were evaluated through a pretested questionnaire. The subject’s dental status was assessed based on four different categories (“fully dentate”, “lost 1–5 teeth”, and “lost > 5 teeth”). Finally, we gathered information on dietary supplement usage (yes/no), oral contraceptives drugs usage (yes/no), and the presence of colitis (yes/no).

### Statistical analysis

In this study, we classified participants according to tertile cut-off points of dTAC score. One-way ANOVA and chi-square tests were used to compare the differences in general characteristics of participants across tertiles of dTAC. We used the analysis of covariance (ANCOVA) test for the comparison of energy-adjusted dietary intakes of participants across tertiles of dTAC. A binary logistic regression test was used to estimate odd ratios and 95% CIs of IBS and its subtypes across tertiles of dTAC in crude and multivariable-adjusted models. In the analyses, models were adjusted for age, sex, energy intake, marital status, education, BMI, physical activity, diabetes history, medication use, smoking, meal regularity, dietary supplements use, chewing sufficiency, frequency of fried food consumption, speed of eating, dental status, intra-meal fluid consumption, and breakfast skipping). We also estimated ORs and 95% CIs for IBS severity (mild, moderate, severe, and very severe) across tertiles of dTAC multivariable ordinal logistic regression. SPSS software (version 24; SPSS Inc.) was used for data analysis and *P* < 0.05 was considered statistically significant.

## Results

In this cross-sectional study, the average age and BMI of the participants were 36.3 ± 7.87 year and 24.9 ± 3.82 kg/m2. In the whole study population, the prevalence of IBS, IBS with constipation (IBS-C), IBS with diarrhoea (IBS-D), mixed IBS (IBS-M), and un-subtyped IBS (IBS-U) were 22.2, 7.5, 4.6, 3.8, and 6.2%, respectively. The dTAC score mean was 7.81 ± 3.45 (mmol/100 gr) and ranged from 0.57 to 23.72. General features of the study participants as well as the rate of the prevalence of IBS and its subtypes across tertiles of dTAC are presented in Table 1. Accordingly, participants in the highest tertile of dTAC score were older (p < 0.001), had higher education levels (p < 0.001), and had greater adherence to a regular meal pattern (p < 0.001) than those in the lowest tertile. We did not find any significant findings in terms of other characteristics across tertiles of dTAC score.

**Table 1:** Baseline Characteristics of study participants as well as the prevalence of IBS and its subtypes across tertiles of dTAC^a^

**Variables**	Tertiles of dTAC			*P* value ^b^
	T1 (< 6.06)	T2 (6.06–8.96)	T3 (> 8.96)	
dTAC	4.30 ± 1.32	7.48 ± 0.83	11.65 ± 2.46	< 0.001
Age (y)	35.6 ± 7.79	36.1 ± 7.92	37.2 ± 7.82	< 0.001
BMI (kg/m2)	24.8 ± 3.92	24.9 ± 3.72	25.0 ± 3.82	0.44
Female (%)	648 (57.9)	641 (57.2)	670 (59.8)	0.44
Married (%)	900 (82.5)	901 (82.0)	889 (80.6)	0.80
Education (university graduated) (%)	637 (56.9)	707 (63.1)	737 (65.7)	< 0.001
Physically active c (%)	136 (12.1)	147 (13.1)	160 (14.3)	0.33
Current smoker (%)	150 (13.4)	150 (13.4)	164 (14.6)	0.62
Regular meal pattern				< 0.001
Never/sometimes	501 (45.7)	419 (37.8)	395 (35.7)	
Often/always	596 (54.3)	689 (62.2)	712 (64.3)	
Chewing sufficiently (a lot)	128 (11.6)	145 (13.1)	158 (14.2)	0.19
Fluid consumption				0.29
< 3 glasses/day	1054 (96.2)	1059 (96.8)	1064 (97.3)	
≥ 3 glasses/day	42 (3.8)	35 (3.2)	29 (2.7)	
Breakfast skipping	89 (8.3)	73 (6.7)	76 (7.0)	0.29
Frequent fried food intake				0.56
≤ 3 times/wk	917 (85.4)	920 (84.6)	909 (83.7)	
> 3 times/wk	157 (14.6)	168 (15.4)	177 (16.3)	
Speed of eating				0.29
< 10 min	109 (9.7)	92 (8.2)	112 (10.0)	
≥ 10 min	1011 (90.3)	1029 (91.8)	1009 (90.0)	
Disease history^d^ (%)	35 (3.1)	34 (3.0)	31 (2.8)	0.87
Medication use^e^ (%)	70 (6.3)	59 (5.3)	75 (6.7)	0.35
Dietary supplement use (%)	339 (30.3)	320 (28.5)	350 (31.2)	0.37
Dental status^f^ (%)	81 (7.4)	77 (7.1)	96 (8.9)	0.57
IBS (%)	256 (22.9)	252 (22.5)	240 (21.4)	0.69
IBS-C (%)	93 (8.3)	73 (6.5)	87 (7.8)	0.26
IBS-D (%)	51 (4.6)	52 (4.6)	53 (4.7)	0.98
IBS-M (%)	46 (4.1)	45 (4.0)	38 (3.4)	0.63
IBS-U (%)	66 (5.9)	82 (7.3)	62 (5.5)	0.18

IBS-C: IBS with constipation; IBS-D: IBS with diarrhea; IBS-M: mixed IBS; IBS-U: unsubtyped IBS

^a^Data are mean ± SD, unless indicated otherwise

^b^Obtained from ANOVA or chi-square test, where appropriate

^c^ ≥ 1 h/week physical activity

^d^Chronic disease included: diabetes and colitis

^e^Medications included omeprazole, pantoprazole, ranitidine, cimetidine, famotidine, clidinium, hyoscine, blandola, dimethicone, digestive, pancreatin, antiacid, diphenoxylate, loperamide, nortriptyline, amitriptyline or imipramine, fluoxetine, citalopram, fluvoxamine, and sertraline

^f^Dental status > 5 teeth lost

Table 2 shows dietary intakes of participants across tertiles of dTAC. A greater dTAC score was significantly associated with higher energy intake, carbohydrates, dietary fibers, saturated fatty acids, some vitamin A, vitamin C, vitamin B6, vitamin B9, calcium, fruits, vegetables, whole grains, tea and coffee, and pickles. It was also significantly associated with lower intakes of protein, fats, vitamin E, vitamin B1, vitamin B12, Fe, zinc, white meat, red and processed meat, and refined grains.

**Table 2:** Dietary intakes of study participants across tertiles of dTAC^a^

	Tertiles of dTAC			*P* value^b^
	T1 (N = 971)	T2 (N = 1008)	T3 (N = 1008)	
Nutrients				
Energy intake (kcal/d)	1842.0 ± 22.6	2426.0 ± 22.1	2859.9 ± 22.2	< 0.001
Carbohydrate (g/d)	280.4 ± 1.64	294.4 ± 1.48	306.8 ± 1.59	< 0.001
Protein g/d)	91.2 ± 0.47	88.3 ± 0.42	85.4 ± 0.46	< 0.001
Fat (g/d)	102.0 ± 0.64	98.2 ± 0.57	95.7 ± 0.62	< 0.001
Fiber (g/d)	20.1 ± 0.19	22.3 ± 0.17	25.3 ± 0.18	< 0.001
SFA (g/d)	18.8 ± 7.61	23.8 ± 9.00	26.9 ± 8.92	0.01
Vitamin A (RAE/d)	480.3 ± 7.00	517.3 ± 6.29	559.6 ± 6.77	< 0.001
Vitamin C (mg/day)	74.5 ± 1.66	99.3 ± 1.50	130.3 ± 1.61	< 0.001
Vitamin D (μg/d)	0.97 ± 0.01	0.96 ± 0.02	0.97 ± 0.02	0.70
Vitamin E (mg/d)	22.7 ± 0.20	21.1 ± 0.18	20.6 ± 0.20	< 0.001
Vitamin B1 (mg/d)	1.86 ± 0.02	1.89 ± 0.02	1.79 ± 0.02	< 0.001
Vitamin B6 (mg/d)	1.94 ± 0.01	1.97 ± 0.01	2.03 ± 0.01	< 0.001
Folate (μg/day)	286.0 ± 2.50	317.1 ± 2.25	358.1 ± 2.42	< 0.001
Vitamin B12 (μg/day)	3.04 ± 0.04	2.97 ± 0.03	2.87 ± 0.03	0.01
Calcium (mg/day)	965.7 ± 14.7	1012.2 ± 13.2	962.5 ± 14.2	0.01
Fe (mg/day)	18.0 ± 0.11	17.8 ± 0.10	17.1 ± 0.11	< 0.001
Zinc (mg/day)	11.3 ± 0.06	11.1 ± 0.05	10.9 ± 0.06	< 0.001
Food groups				
Fruits (g/d)	177.3 ± 7.07	301.9 ± 6.35	469.0 ± 6.84	< 0.001
Vegetables (g/d)	219.7 ± 4.07	237.5 ± 3.65	259.9 ± 3.93	< 0.001
White meat (g/d)	69.4 ± 1.51	62.6 ± 1.36	58.9 ± 1.46	< 0.001
Red and processed meat (g/d)	92.6 ± 1.55	82.7 ± 1.39	78.08 ± 1.49	< 0.001
Nuts, legumes and soy (g/d)	59.1 ± 1.26	56.0 ± 1.13	56.6 ± 1.22	0.18
Refined grains (g/d)	438.9 ± 5.60	403.2 ± 5.03	338.2 ± 5.41	< 0.001
Whole grains (g/d)	34.1 ± 2.62	42.2 ± 2.36	51.1 ± 2.54	< 0.001
Dairy intake (g/d)	331.6 ± 9.10	356.6 ± 8.20	357.5 ± 8.80	0.09
Tea and coffee (g/d)	158.0 ± 8.08	355.7 ± 7.26	625.3 ± 7.82	< 0.001
Pickles (g/d)	7.10 ± 0.61	8.90 ± 0.55	10.84 ± 0.59	< 0.001

^a^Data are means ± standard error (SE), unless indicated

^b^All values were adjusted for age, sex and energy, except for dietary energy intake, which was only adjusted for age and sex using ANCOVA

Multivariable-adjusted ORs and 95% CIs for IBS across tertile categories of dTAC were shown in Table 3. In crude model, the results did not show any significant association between dTAC and odds of IBS among whole population (OR 0.92; 95% CI (0.75–1.12); P_trend_ = 0.41). After adjustment for confounders in different models, the results remained non-significant. Stratified analyses based on the gender ((male: OR 0.94; 95% CI (0.67–1.31); P trend = 0.73), female: (OR 0.90; 95% CI (0.75–1.24); P_trend_ = 0.39)) and BMI ((BMI < 25 (kg/m^2^): OR 0.80; 95% CI (0.60–1.42); P_trend_ = 0.13), BMI˃25 (kg/m^2^): (OR 1.05; 95% CI (0.77–1.42); P_trend_ = 0.75)) also did not reveal any significant associations in crude model. These findings also remained non-significant even after adjustment for potential confounders.

**Table 3:** Gender- and BMI-stratified ORs and 95% CIs for IBS across tertiles of dTAC

	Tertiles of dTAC			P_trend_
	T1 (< 6.06)	T2 (6.06–8.96)	T3 (> 8.96)	
	OR	OR (95% CI)	OR (95% CI)	
Whole population				
Crude	1.00	0.98 (0.80–1.19)	0.92 (0.75–1.12)	0.41
Model 1^a^	1.00	1.08 (0.87–1.36)	1.12 (0.88–1.44)	0.35
Model 2	1.00	1.11 (0.88–1.39)	1.11 (0.86–1.43)	0.43
Model 3	1.00	1.15 (0.90–1.46)	1.12 (0.86–1.46)	0.42
Model 4	1.00	1.09 (0.85–1.39)	1.07 (0.81–1.40)	0.64
Male				
Crude	1.00	1.01 (0.73–1.39)	0.94 (0.67–1.31)	0.73
Model 1^b^	1.00	1.33 (0.90–1.97)	1.40 (0.91–2.15)	0.13
Model 2	1.00	1.28 (0.86–1.91)	1.31 (0.85–2.03)	0.23
Model 3	1.00	1.56 (1.02–2.39)	1.52 (0.95–2.43)	0.09
Model 4	1.00	1.47 (0.95–2.27)	1.39 (0.86–2.24)	0.21
Female				
Crude	1.00	0.97 (0.75–1.24)	0.90 (0.75–1.24)	0.39
Model 1^b^	1.00	0.99 (0.75–1.30)	1.01 (0.75–1.37)	0.94
Model 2	1.00	1.03 (0.78–1.36)	1.01 (0.74–1.38)	0.95
Model 3	1.00	1.01 (0.75–1.35)	0.96 (0.69–1.34)	0.83
Model 4	1.00	0.96 (0.71–1.29)	0.95 (0.68–1.32)	0.77
BMI < 25 (kg/m^2^)				
Crude	1.00	1.09 (0.83–1.42)	0.80 (0.60–1.42)	0.13
Model 1^a^	1.00	1.19 (0.87–1.62)	1.02 (0.72–1.44)	0.91
Model 2	1.00	1.21 (0.88–1.66)	1.01 (0.71–1.43)	1.00
Model 3	1.00	1.19 (0.85–1.66)	1.00 (0.69–1.45)	0.97
BMI ≥ 25 (kg/m^2^)				
Crude	1.00	0.90 (0.66–1.23)	1.05(0.77–1.42)	0.75
Model 1^a^	1.00	1.04 (0.73–1.47)	1.25 (0.85–1.83)	0.25
Model 2	1.00	1.03 (0.72–1.47)	1.21 (0.82–1.79)	0.33
Model 3	1.00	1.13 (0.78–1.63)	1.25 (0.82–1.88)	0.30

Model 1^a^: adjusted for age, gender, and energy intake

Model 1^b^: adjusted for age

Model 2: further adjusted for physical activity, marital status, education level, smoking, chronic disease, medication use and dietary supplement intake

Model 3: further adjusted for regular meal pattern, eating rate, chewing sufficiency, breakfast skipping, fluid consumption, fried food intake, and dental status

Model 4: additionally, adjusted for BMI

The results of the analyses on the association between dTAC and odds of IBS severity are provided in Table 4. Accordingly, being in the third compared with the first tertile of dTAC was not significantly associated with odds of IBS severity in the crude model among the whole population (OR 0.88; 95% CI (0.67–1.15). Our analyses also failed to show any significant association when various confounders were controlled for. No significant associations were found when the analyses stratified by gender ((male: OR 0.90; 95% CI (0.56–1.45), female: (OR 0.87; 95% CI (0.63–1.20)) and BMI ((BMI < 25 (kg/m^2^): (OR 0.91; 95% CI (0.63–1.33), BMI ≥ 25 (kg/m^2^): (OR 0.77; 95% CI (0.51–1.17)) in crude model. When potential confounders were controlled for, the results were similar.

**Table 4:** Gender- and BMI-stratified ORs and 95% CIs for IBS severity across tertiles of dTAC

	Tertiles of dTAC		
	T1 (< 6.06)	T2 (6.06–8.96)	T3 (> 8.96)
	OR	OR (95% CI)	OR (95% CI)
Whole population			
Crude	1.00	0.90 (0.69–1.17)	0.88 (0.67–1.15)
Model 1^a^	1.00	0.86 (0.64–1.16)	0.86 (0.62–1.19)
Model 2	1.00	0.90 (0.66–1.21)	0.83 (0.60–1.16)
Model 3	1.00	0.93 (0.67–1.28)	0.87 (0.61–1.25)
Model 4	1.00	0.93 (0.67–1.27)	0.82 (0.57–1.18)
Male			
Crude	1.00	1.10 (0.70–1.74)	0.90 (0.56–1.45)
Model 1^b^	1.00	0.98 (0.58–1.68)	0.85 (0.47–1.53)
Model 2	1.00	0.90 (0.52–1.56)	0.74 (0.40–1.36)
Model 3	1.00	0.94 (0.51–1.74)	0.74 (0.38–1.46)
Model 4	1.00	0.88 (0.47–1.65)	0.73 (0.90–1.47)
Female			
Crude	1.00	0.81 (0.58–1.22)	0.87 (0.63–1.20)
Model 1^b^	1.00	0.80 (0.56–1.14)	0.85 (0.57–1.26)
Model 2	1.00	0.88 (0.61–1.26)	0.84 (0.56–1.25)
Model 3	1.00	0.89 (0.61–1.31)	0.92 (0.60–1.41)
Model 4	1.00	0.91 (0.62–1.34)	0.85 (0.55–1.32)
BMI < 25 (kg/m^2^)			
Crude	1.00	0.96 (0.67–1.39)	0.91 (0.63–1.33)
Model 1^a^	1.00	0.85 (0.56–1.29)	0.85 (0.54–1.33)
Model 2	1.00	0.94 (0.61–1.43)	0.82 (0.52–1.30)
Model 3	1.00	1.14 (0.72–1.79)	0.96 (0.59–1.58)
BMI ≥ 25 (kg/m^2^)			
Crude	1.00	0.81 (0.54–1.21)	0.77 (0.51–1.17)
Model 1^a^	1.00	0.80 (0.51–1.25)	0.81 (0.49–1.35)
Model 2	1.00	0.76 (0.48–1.22)	0.83 (0.49–1.39)
Model 3	1.00	0.69 (0.42–1.14)	0.73 (0.42–1.30)

Model 1^a^: adjusted for age, gender, and energy intake

Model 1^b^: adjusted for age

Model 2: further adjusted for physical activity, marital status, education level, smoking, chronic disease, medication use and dietary supplement intake

Model 3: further adjusted for regular meal pattern, eating rate, chewing sufficiency, breakfast skipping, fluid consumption, fried food intake, and dental status

Model 4: additionally, adjusted for BMI

In Table 5 ORs and 95% CIs for IBS subtypes across tertiles of dTAC were presented. In crude model, there was no significant associations between dTAC and odds of IBS-C (OR 0.93; 95% CI (0.68–1.26); P_trend_ = 0.63), IBS-D (OR 1.04; 95% CI (0.70–1.54); P_trend_ = 0.84), IBS-M (OR 0.82; 95% CI (0.53–1.27); P_trend_ = 0.38), and IBS-U (OR 0.93; 95% CI (0.65–1.34); P_trend_ = 0.72), respectively. We could not see any significant association even after adjustment for potential confounders.

**Table 5:** Crude and multivariable-adjusted ORs and 95% CIs for IBS subtypes across tertiles of dTAC

	Tertiles of dTAC			P_trend_
	T1 (< 6.06)	T2 (6.06–8.96)	T3 (> 8.96)	
	OR	OR (95% CI)	OR (95% CI)	
IBS-C				
Crude	1.00	0.77 (0.56–1.06)	0.93 (0.68–1.26)	0.63
Model 1	1.00	0.75 (0.52–1.08)	0.99 (0.68–1.45)	0.99
Model 2	1.00	0.76 (0.52–1.09)	0.95 (0.64–1.40)	0.82
Model 3	1.00	0.73 (0.50–1.07)	0.92 (0.62–1.39)	0.71
Model 4	1.00	0.69 (0.46–1.01)	0.90 (0.60–1.37)	0.64
IBS-D				
Crude	1.00	1.02 (0.69–1.51)	1.04 (0.70–1.54)	0.84
Model 1	1.00	1.24 (0.79–1.96)	1.39 (0.85–2.28)	0.19
Model 2	1.00	1.23 (0.78–1.96)	1.42 (0.86–2.33)	0.17
Model 3	1.00	1.35 (0.84–2.19)	1.52 (0.90–2.56)	0.12
Model 4	1.00	1.39 (0.86–2.25)	1.43 (0.84–2.43)	0.20
IBS-M				
Crude	1.00	0.98 (0.64–1.48)	0.82 (0.53–1.27)	0.38
Model 1	1.00	0.95 (0.59–1.51)	0.89 (0.53–1.50)	0.66
Model 2	1.00	0.95 (0.59–1.52)	0.89 (0.52–1.51)	0.66
Model 3	1.00	1.16 (0.70–1.92)	1.08 (0.61–1.90)	0.80
Model 4	1.00	1.11 (0.67–1.85)	1.01 (0.57–1.79)	0.98
IBS-U				
Crude	1.00	1.26 (0.90–1.76)	0.93 (0.65–1.34)	0.72
Model 1	1.00	1.56 (1.06–2.27)	1.21 (0.78–1.87)	0.42
Model 2	1.00	1.60 (1.09–2.34)	1.19 (0.76–1.85)	0.46
Model 3	1.00	1.53 (1.03–2.29)	1.07 (0.67–1.72)	0.78
Model 4	1.00	1.43 (0.95–2.15)	1.05 (0.65–1.70)	0.72

IBS-C: IBS with constipation; IBS-D: IBS with diarrhea; IBS-M: mixed IBS; IBS-U: unsubtyped IBS

Model 1: adjusted for age, gender, and energy intake

Model 2: further adjusted for physical activity, marital status, education level, smoking, chronic disease, medication use and dietary supplement intake

Model 3: further adjusted for regular meal pattern, eating rate, chewing sufficiency, breakfast skipping, fluid consumption, fried food intake, and dental status

Model 4: additionally, adjusted for BMI

## Discussion

In the current study, we failed to find a significant association between dTAC and odds of IBS in both crude and adjusted models. The results also did not show any associations between dTAC and odds of the severity of the disease and its subtypes. Moreover, no significant associations were found even after stratification by gender and BMI which altogether point out the fact that there may be no associations between the overall antioxidant capacity of the diet and odds of IBS and IBS severity.

The IBS as a multifactorial painful chronic GI disorder is identified by alterations in bowel habits [51]. It has been found that inflammation may play a role in visceral hypersensitivity, a condition that leads to pain and discomfort in patients with IBS [4]. In this line, a number of studies showed that IBS patients had higher levels of pro-inflammatory cytokines than healthy control subjects [52, 53]. On the other side, additionally, inflammation and oxidative stress are linked, since leukocytes produce reactive oxygen species (ROS) when they are activated by resident cells (endothelial and smooth muscle cells) [54]. The results of previous research demonstrated the altered oxidant-antioxidant balance in patients with IBS compared to the healthy controls so that in those studies, the serum levels of prooxidant compounds increased and the serum levels of antioxidant compounds decreased [7, 53]. Nevertheless, the results from our study showed that dietary antioxidants were not significantly associated with the odds of IBS and its severity in a large sample size study among Iranian adults. Although there is no similar study that directly addressed such an association, however, other studies have investigated the associations between dTAC and other GI disorders and showed inconsistent results [38, 55–57]. Accordingly, The highest versus lowest quartile of dTAC was associated with a reduced risk of ulcerative colitis in a case–control study involving 62 IBD patients and 124 healthy controls from the Iranian population [38]. The design of that study was different, and the analyses were not controlled for dietary antioxidant supplements as an important source of antioxidants. The Evidence of European Prospective Investigation into Cancer (EPIC) study on 521,457 subjects from 10 European countries showed that the highest versus lowest quintile of both FRAP and TRAP, as indicators of dietary antioxidant capacity, was significantly associated with a reduction in the risk of gastric cancer [57]. A case–control study including 1953 patients with colorectal cancer and 4,154 controls demonstrated an inverse association between FRAP, TEAP, and TRAP with the risk of colorectal cancer in Italian populations [56]. Nevertheless, the results of the Health Professionals Follow-up Study showed that dTAC was not significantly associated with colorectal or colon cancer but was inversely associated with the risk incidence of rectal cancer. Interestingly, total antioxidant capacity (from both foods and supplements) was not associated with colorectal, colon, and rectal cancer [55]. Taken tighter, due to the lack of similar studies among other populations and the complex nature of the IBS disease, there are limited data to establish the potential contribution of dTAC to IBS severity and odds. In addition, the results of previous research pointed out the fact that there is little evidence about the inadequacy of single antioxidant-rich food or nutrient in patients with IBS. For example, the results of a case–control study (187 IBS patients and 374 age and gender-matched controls) among the Sweden population showed that IBS patients had a significantly higher intake of vitamin C, B9, Iron, vitamin E, and dietary fiber; as well as lower intake of B2, vitamin A, potassium, and calcium compared to the control group. This study also demonstrated that daily nutrient intake in IBS patients met national nutrient recommendations and no association was found between the nutrient intake and IBS subtypes or IBS symptom severity [9]. This finding also has been proven in previous research [14, 58–60] and is against the findings of the research that indicated that dietary restrictions could lead to nutrient deficiencies among IBS patients [12, 61, 62]. Our results about the non-significant association between dTAC and odds of IBS may also confirm by studies that evaluated serum oxidative stress markers [53, 63]. Interestingly, research found that inflammatory cytokines and oxidative stress biomarkers had a significant and non-significant association with digestive symptoms and quality of life in 90 IBS patients and 90 gender- and age-matched healthy controls [53]. A review was also conducted on available data to explore the possible relevance of oxidative stress status in the pathogens of IBS. The authors concluded that there is a possible correlation between some complications such as mild inflammatory patterns, neurological impairment, emotional over-responsiveness, and oxidative stress in IBS patients which are not followed by tissue destruction. Moreover, they suggested that it is not yet clear whether neurological inflammation, impairments, or oxidative stress are key determinants or in which way these three interact in IBS pathology and there is a need to find possible explanations for the occurrence of oxidative imbalances and its role in the pathogenesis of IBS [63]. So, our results along with reviewing the available data indicate that there is still a need for a more comprehensive perspective or clinical trial studies to elucidate the potential role of antioxidants especially dietary ones in the prevention and management of IBS.

We did not find any significant associations between the dTAC and IBS when the analyses stratified by sex. There is evidence that IBS is more prevalent in women than men [64]. This might be because of modulatory effects sex hormones. It was suggested that sex hormones may involve in the onset or exacerbation of IBS. It was proposed that slow GI transit, reduced colonic transit time or delayed gastric empty might be mediated by sex hormones [65].

Our results did not show any significant associations between the dTAC and IBS when the analyses stratified based on BMI. Previous research showed that IBS might be differed based on the body weight differences. Accordingly, a cross-sectional study showed a significant positive association between obesity and IBS symptoms [66]. This also confirmed in another study, too [67]. It has been suggested that overweight/obesity are associated with elevated levels of inflammatory markers [68]. So, high prevalence of IBS in overweight/obese subjects might also be explained by inflammation [69].

This cross-sectional study has several strengths. This was the first study investigating the association between dTAC and odds of IBS and its severity. The study design was based on the general population and its sample size was high which reduces the risk of selection bias and the risk of type II error and increases internal validity, respectively. Because the study was conducted on a general population, it included subjects of all grades of IBS who visited or did not visit a doctor. Since there were heterogeneous characteristics within the IBS population in this study, the results cannot be compared with those of studies involving merely referred participants. We also controlled the analyses for several confounders. Another advantage of the study is the use of the FRAP index to estimate dTAC as the FRAP test is the only one that directly measures antioxidants in food samples. In some assays, the measurement of inhibition depends on the kind of reactive species used in the reaction mixture, while in other assays, the inhibition is dependent on free radicals generated in the reaction mixture. So, they indirectly estimate dTAC [70].

Our study similar to any other study has also had some limitations that should be taken into account. This had a cross-sectional design thus we can't infer the causality of possible relationships between dTAC and IBS. Information about the IBS and its severity was obtained through the Rome III questionnaire. It is possible that misclassification has not been eliminated despite the validity of the questionnaire among Iranian adults. The participants were from Isfahan province; thus, the results should be cautiously generalized to other populations in Iran. We had no information about the FRAP values of local foods in Iran. Accordingly, this information was derived from international databases which were different from Iranian foods. Finally, Despite dietary FRAP scores being able to provide an advantage over other indices, previous studies did not confirm their relationship with plasma FRAP measurements [25, 26]. These findings may be justified by differences in endogenous antioxidant homeostasis and factors that affect dietary antioxidants absorption or metabolism [71].

## Conclusion

The present study indicates that dTAC may not be associated with the odds of occurrence of IBS and its severity. In addition, by reviewing previous research we could not infer the possible roles of dietary or plasma antioxidants in the pathogenesis of IBS. So, further prospective studies or clinical trials can help to elucidate the existence of any possible associations.

## Data Availability

Datasets used and/or analyzed in the study are available from the corresponding author on reasonable request.

## Abbreviations

ANCOVA: Analysis of covariance
BMI: Body mass index
dTAC: Dietary total antioxidant capacity
EPIC: Evidence of European Prospective Investigation into Cancer
SEPAHAN: Epidemiology of Psychological, Alimentary Health and Nutrition
FRAP: Ferric-reducing antioxidant power
GPPAQ: General Practice Physical Activity Questionnaire
GI: Gastrointestinal
IBS: Irritable bowel syndrome
IUMS: Isfahan University of Medical Sciences
DS-FFQ: Semi-quantitative validated food frequency questionnaire
